# Not all patients benefit from the postoperative antifibrinolytic treatment: clinical evidence against the universal use of tranexamic acid following total knee arthroplasty

**DOI:** 10.1186/s13018-022-02958-0

**Published:** 2022-01-29

**Authors:** Jiacheng Liu, Han Wang, Xiangdong Wu, Yiting Lei, Wei Huang

**Affiliations:** 1 Department of Orthopedics, Orthopedic Laboratory of Chongqing Medical University, The First Affiliated Hospital of Chongqing Medical University, Chongqing, 400016 China; 2 Department of Orthopedics, Zhangzhou Affiliated Hospital of Fujian Medical University, Zhangzhou, 363000 Fujian China; 3 Department of Orthopedic Surgery, Peking Union Medical College Hospital, Chinese Academy of Medical Sciences and Peking Union Medical College, Beijing, 100730 China

**Keywords:** Total knee arthroplasty, Fibrinolytic shutdown, Tranexamic acid, Antifibrinolysis

## Abstract

**Background:**

The empirical use of tranexamic acid (TXA) for bleeding remains controversial because of the distinct fibrinolytic phenotypes observed after injury. This study sought to assess the efficacy of postoperative TXA in patients presenting with different fibrinolytic phenotypes after total knee arthroplasty (TKA).

**Methods:**

This retrospective study included 270 patients who underwent primary TKA. The patients were divided into two groups: Group A, received no postoperative TXA, and Group B, received postoperative TXA; they were further categorized into four subgroups based on postoperative fibrinolytic phenotypes (non-fibrinolytic shutdown [NFSD] and fibrinolytic shutdown [FSD]). Fibrinolytic phenotypes were determined using percentage of clot lysis 30 min after maximum strength (LY30) level measured on postoperative day 1 (POD1). Data on perioperative hidden blood loss (HBL), decrease in the hemoglobin level (ΔHb), allogeneic blood transfusion (ABT) rate, fibrin degradation product (FDP) level, D-dimer (D-D) level, prothrombin time (PT), and activated partial thromboplastin time (APTT) as well as clinical baseline data were collected and compared.

**Results:**

No differences in baseline clinical data were noted. Among patients presenting with NFSD, those in Group B had significantly lower HBL and ΔHb on POD1 and POD3 than those in Group A. Among patients presenting with FSD, perioperative HBL and ΔHb were similar between the two groups. No differences were observed in perioperative ABT rate, FDP level, D-D level, PT, and APTT.

**Conclusions:**

Patients exhibit various fibrinolytic phenotypes after TKA. Postoperative antifibrinolytic strategies may be beneficial for patients presenting with NFSD, but not for those presenting with FSD. The LY30 level may guide targeted TXA administration after TKA. However, well-designed prospective randomized controlled trials are needed to obtain more robust data.

## Background

Total knee arthroplasty (TKA) is usually performed to treat end-stage knee arthritis [1]. However, this surgical procedure has been associated with postoperative hidden blood loss (HBL) [2]. Tranexamic acid (TXA) effectively reduces HBL after TKA and is thus recommended for use as a routine antifibrinolytic agent in all TKA surgeries [3]. Robust data from several large-scale multicenter randomized controlled trials (RCTs) have indicated that the timing is crucial and TXA should be administered soon after the onset of bleeding [4–6]. Moreover, TXA should be empirically used in all cases of bleeding to reduce blood loss and decrease mortality [4].

Nevertheless, recent evidence from the cases of traumatic and acute bleeding warns against the universal use of TXA after injury [7]. A previous study has demonstrated various fibrinolytic phenotypes after injury and suggested that clinicians should not focus on the timing alone when considering TXA administration [8]. Approximately 46% of patients reportedly present with pathologically downregulated fibrinolysis after injury, which is known as fibrinolytic shutdown (FSD) [9]. Functioning as an antifibrinolytic agent, TXA inhibits the activity of the fibrinolytic system, stabilizes the formed clots that seal the broken vessels, and effectively reduces blood loss secondary to hyperfibrinolysis. Therefore, researchers argue that TXA may be administered with caution in bleeding patients presenting with FSD, as there was nothing to inhibit in fact, and this phenotype has been associated with an increased risk of venous thromboembolism (VTE) and postinjury mortality [10, 11].

As mentioned earlier, TXA administration after TKA has been demonstrated to be beneficial for the patients [2]. However, the aforementioned recent evidence poses some important questions: (1) Do patients exhibit similar but different fibrinolytic phenotypes after TKA? and (2) do all patients with different fibrinolytic phenotypes benefit from antifibrinolytic therapy after TKA? Therefore, this study aimed to evaluate the efficacy of TXA in patients presenting with distinct fibrinolytic phenotypes after TKA.

## Methods

### Study design

This is a retrospective study. All patients consecutively admitted to our center between September 2016 and November 2019 for TKA were screened for eligibility. All eligible patients received 1.5 g of intravenous TXA 30 min before incision and 1 g of topical TXA before wound closure during TKA. Patients who received no postoperative TXA constituted Group A, whereas those who received postoperative TXA (1 g of intravenous TXA at 3, 12, 24, 48, and 72 h after surgery) constituted Group B. The two groups were further divided into a total of four subgroups based on postoperative fibrinolytic phenotypes (non-FSD [NFSD] or FSD): (1) NFSD, percentage of clot lysis 30 min after maximum strength (LY30) ≥ 0.8% on postoperative day 1 (POD1), and (2) FSD, LY30 < 0.8% on POD1.

### Eligibility criteria

We applied strict inclusion and exclusion criteria to control the potential heterogeneity among the patients. Table 1 presents the specific inclusion and exclusion criteria.

**Table 1:** The inclusion and exclusion criteria

Inclusion criteria	Exclusion criteria
1. Receiving unilateral primary TKA due to end-stage knee diseases	1. UKA
2. Receiving pre- and intraoperative TXA or pre-, intra-, and postoperative TXA	2. Bilateral TKA
3. Preoperative hemoglobin level > 90 g/L	3. Tumor-related TKA
	4. Revision TKA
	5. Combined with significant hepatic or renal dysfunction
	6. Combined with coagulation dysfunction preoperatively
	7. Combined with knee infection preoperatively

*TKA* total knee arthroplasty, *TXA* tranexamic acid, *UKA* unilateral knee arthroplasty

### Surgical procedure

Under general anesthesia, all patients underwent TKA performed by the same surgical team comprising three senior orthopedists. All TKA surgeries were performed following the paramedian approach with patients in the supine position. Pneumatic tourniquet with a set pressure of 30 mmHg was routinely applied throughout the duration of surgery.

### Postoperative management

The indication for perioperative allogeneic blood transfusion (ABT) at our center is follows: (1) hemoglobin level < 70 g/L, with or without anemia symptoms, or (2) 70 g/L < hemoglobin level < 100 g/L, with anemia symptoms. As thromboprophylaxis, 4000 IU of low molecular weight heparin or 10 mg of rivaroxaban was administered daily after surgery. Patient-controlled analgesia and nerve block combined with selective cyclooxygenase-2 inhibitors (i.e., celecoxib and etoricoxib) were used for postoperative pain management. As postoperative infection prophylaxis, 1.5 g of cefuroxime sodium was administered twice daily from POD1 to POD3.

### Outcome assessments

The primary outcomes of this study included HBL and decrease in the hemoglobin level (ΔHb). The former was calculated using formulas reported by Gross and Nadler [12, 13]. The latter was calculated using perioperatively monitored hemoglobin levels. Moreover, the fibrin degradation product (FDP) level, D-dimer (D-D), prothrombin time (PT), and activated partial thromboplastin time (APTT) were determined as secondary outcomes. During their hospital stay, the aforementioned parameters of the patients were routinely evaluated on POD1, POD3, POD5, and POD7 at the Department of Clinical Laboratory of our medical center. In addition, data on other clinical characteristics such as sex, age, body mass index (BMI), and surgery duration were also collected and compared.

### Thromboelastography

Thromboelastography (TEG) includes seven parameters: (1) reaction time (R), period to 2 mm amplitude, representing enzymatic reaction function; (2) kinetics (K), period from 2 to 20 mm amplitude, representing clot kinetics; (3) alpha angle (α-angle), slope between R and K, representing fibrinogen level; (4) maximum amplitude (MA), representing maximum platelet function; (5) percentage of clot lysis 30 min after MA (LY30), representing fibrinolytic activity; (6) estimated percent lysis within 30 min after MA, representing fibrinolytic activity; and (7) comprehensive coagulation index, representing a linear combination of R, K, α-angle, and MA values. TEG was performed using TEG® Hemostasis Analyzer, Model 5000 (Haemonetics Corporation, Braintree, MA, USA).

### Sample size calculation

Sample size was determined using the PASS (version 11; NCSS, LLC. Kaysville, UT, USA) software. According to a previously published study [2], the maximum ΔHb was 15.79 g/L after administration of a dosage of 1.5 g intravenous TXA 30 min before skin incision, 1 g of intra-articular TXA before wound closure, and 1 g of TXA at 3, 12, 24, 48, and 72 h after surgery. To detect a difference of 4 g/L in the maximum ΔHb, with a power of 0.90 and significance level of 0.05, 46 patients were needed per arm.

### Statistical analysis

Excel (Microsoft Corporation, WA, USA) was used to collect and manage the study data, and SPSS version 24.0 (IBM Corporation, Armonk, NY, USA) was used to perform data analyses. The quantitative data were presented as mean ± standard deviation, whereas the qualitative data were presented as frequencies with percentages. Independent *t* test was used to detect the differences in normally distributed numerical values, whereas the Mann–Whitney *U* test was used to detect the differences in non-normally distributed numerical values. For qualitative parameters, Pearson's Chi-square or Fisher’s exact test was used. Statistical significance was set at a *p* value of < 0.05.

## Results

### Participant flow and baseline characteristics

**Fig. 1:**
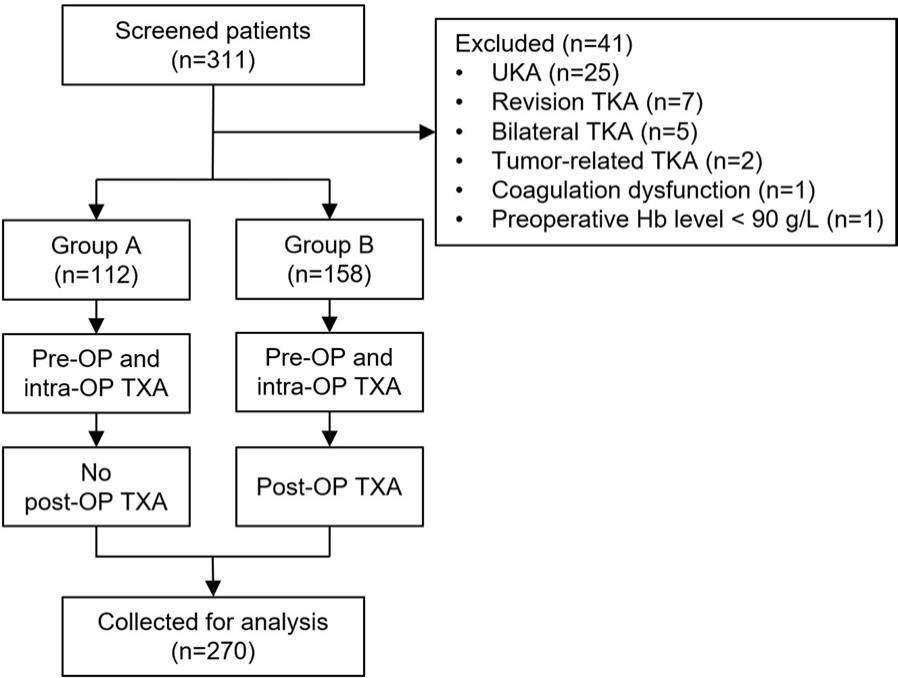
The flowchart of study enrolment. *Pre-OP* preoperative, *Intra-OP* intraoperative, *TXA* tranexamic acid, *Post-OP* postoperative, *UKA* unilateral knee arthroplasty, *TKA* total knee arthroplasty, *Hb* hemoglobin

Figure 1 shows a flowchart of this study. A total of 311 patients who underwent TKA during the study period and received the specified antifibrinolytic therapy were screened for enrollment; among them, 270 were eligible for study inclusion. Table 2 presents clinical baseline data and surgery-related characteristics. The two studied groups were comparable with respect to sex, age, BMI, major diagnosis, surgical site, or other clinical baseline data.

**Table 2:** Clinical and surgery-related baseline data between study groups

	Group A (*n* = 112)	Group B (*n* = 158)	*p*
Female, *n* (%)	93 (83.04%)	129 (81.65%)	0.768^┼^
Age ± SD (year)	68.78 ± 7.60	67.23 ± 8.72	0.132*
Height ± SD (m)	1.56 ± 0.06	1.57 ± 0.06	0.185*
Weight ± SD (kg)	63.64 ± 9.50	61.84 ± 9.53	0.143*
BMI ± SD (kg/m^2^)	26.05 ± 3.88	25.12 ± 3.83	0.074^†^
Major diagnosis			
KOA, *n* (%)	103 (91.96%)	140 (88.61%)	0.365^┼^
KRA, *n* (%)	9 (8.04%)	18 (11.39%)	0.365^┼^
Left TKA, *n* (%)	55 (49.11%)	80 (50.63%)	0.805^┼^
ABT, *n* (%)	3 (2.68%)	6 (3.80%)	0.740^┼^
Intraoperative blood loss ± SD (mL)	65.41 ± 47.31	58.24 ± 59.89	0.295*
Operation time ± SD (min)	94.27 ± 21.38	92.19 ± 24.70	0.473*
LOS ± SD (day)	11.47 ± 3.34	12.22 ± 3.90	0.191*
Post-OP LOS ± SD (day)	6.17 ± 2.69	6.89 ± 3.29	0.101*

*SD* standard deviation, *BMI* body mass index, *KOA* knee osteoarthritis, *KRA* knee rheumatoid arthritis, *TKA* total knee arthroplasty, *ABT* allogeneic blood transfusion, *LOS* length of stay, *Post-OP* postoperative

*Independent-samples *t* test

^┼^Chi-square test

^†^Mann–Whitney *U* test

### Primary outcomes

**Fig. 2:**
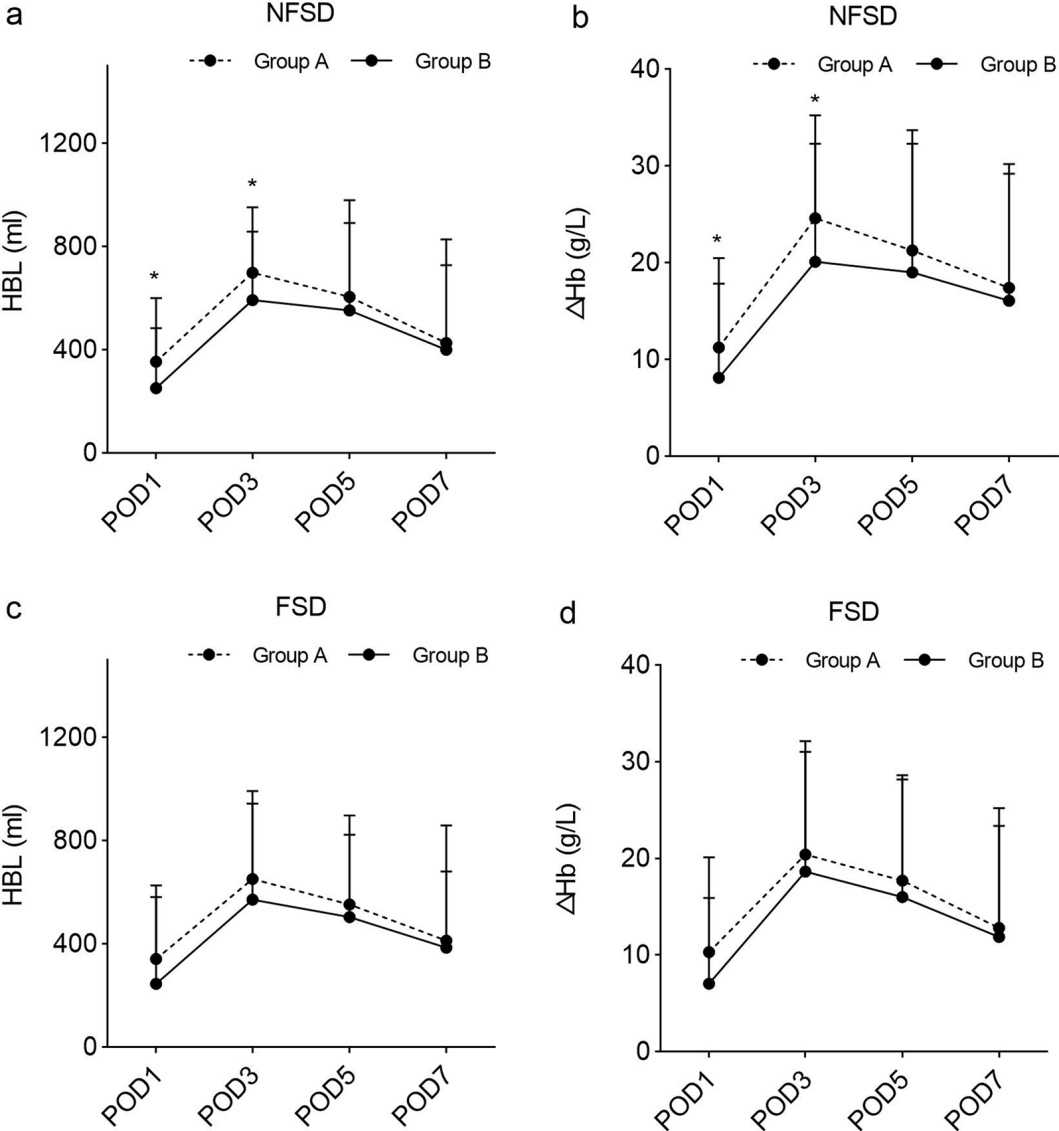
Postoperative trends of hidden blood loss and decline of hemoglobin level. **a** In patients presented with non-fibrinolytic shutdown, postoperative hidden blood loss between Group A and Group B; **b** in patients presented with non-fibrinolytic shutdown, postoperative decline of hemoglobin level between Group A and Group B; **c** in patients presented with fibrinolytic shutdown, postoperative hidden blood loss between Group A and Group B; **d** in patients presented with fibrinolytic shutdown, postoperative decline of hemoglobin level between Group A and Group B. *NFSD* non-fibrinolytic shutdown, *FSD* fibrinolytic shutdown, *HBL* hidden blood loss, *POD* postoperative day, *ΔHb* decline of hemoglobin level; *p value < 0.05

Among patients presenting with NFSD after TKA, those in Group B had significantly lower HBL than those in Group A on POD1 and POD3 (*p* = 0.016 and *p* = 0.021, respectively). Similarly, patients in Group B had significantly lower ΔHb than those in Group A on POD1 and POD3 (*p* = 0.036, *p* = 0.014). However, no difference in HBL and ΔHb was detected between the two groups among patients who presented with FSD after TKA. Figure 2 shows the postoperative trends in HBL and ΔHb between the study groups, and Table 3 summarizes the exact values.

**Table 3:** The post-operative HBL and ΔHb

Outcomes	NFSD (*n* = 95)			FSD (*n* = 175)		
	Group A (*n* = 49)	Group B (*n* = 46)	*p*	Group A (*n* = 63)	Group B (*n* = 112)	*p*
HBL ± SD (mL)						
POD1	354.02 ± 246.50	251.55 ± 244.43	0.016*§	341.84 ± 284.76	246.08 ± 336.03	0.164^†^
POD3	699.30 ± 254.39	592.57 ± 265.40	0.021*§	651.63 ± 293.24	571.39 ± 422.19	0.799*
POD5	605.87 ± 375.22	553.56 ± 338.34	0.512*	522.61 ± 270.32	504.23 ± 394.51	0.423*
POD7	426.59 ± 402.03	400.34 ± 327.94	0.844*	413.04 ± 267.40	386.00 ± 473.98	0.395*
ΔHb ± SD (g/L)						
POD1	11.26 ± 9.24	8.10 ± 9.76	0.036*§	10.33 ± 9.78	7.04 ± 8.87	0.089*
POD3	24.62 ± 10.62	20.12 ± 12.18	0.014*^§^	20.41 ± 10.63	18.64 ± 13.53	0.482*
POD5	21.27 ± 11.05	19.00 ± 14.73	0.911*	17.72 ± 10.88	16.00 ± 12.17	0.808*
POD7	17.42 ± 11.79	16.07 ± 14.15	0.926*	12.82 ± 10.56	11.89 ± 13.33	0.539*

*NFSD* non-fibrinolytic shutdown, *FSD* fibrinolytic shutdown, *HBL* hidden blood loss, *SD* standard deviation, *POD* postoperative day, *ΔHb* decline of hemoglobin level

*Independent-samples *t* test

^†^Mann–Whitney *U* test

^§^Statistically different

### Secondary outcomes

Table 4 presents the exact values of the following parameters: FDP level, D-D level, PT, and APTT. Although enrolled patients appeared to present with different fibrinolytic phenotypes, no significant differences in the aforementioned coagulation and fibrinolysis parameters were observed between the study groups.

**Table 4:** The peri-operative values of FDP, D-D, PT, and APTT

Outcomes	NFSD (*n* = 95)			FSD (*n* = 175)		
	Group A (*n* = 49)	Group B (*n* = 46)	*p*	Group A (*n* = 63)	Group B (*n* = 112)	*p*
FDP ± SD (μg/mL)						
Pre-OP	3.38 ± 5.38	2.20 ± 3.18	0.290*	2.91 ± 4.64	2.27 ± 4.35	0.436*
POD1	19.13 ± 17.82	15.32 ± 11.68	0.314*	21.86 ± 25.87	16.70 ± 10.61	0.209*
POD3	6.82 ± 2.72	6.57 ± 4.72	0.796*	7.29 ± 4.91	7.17 ± 4.58	0.889*
POD5	11.53 ± 6.58	8.41 ± 3.34	0.139*	11.74 ± 6.02	10.98 ± 4.53	0.597*
POD7	13.99 ± 5.70	12.41 ± 3.97	0.422*	13.69 ± 4.34	13.60 ± 5.92	0.963*
D-D ± SD (mg/L)						
Pre-OP	1.21 ± 2.08	0.70 ± 0.86	0.218*	1.08 ± 1.77	0.84 ± 1.65	0.462*
POD1	8.16 ± 7.73	5.73 ± 3.99	0.125*	6.40 ± 3.79	6.14 ± 5.55	0.777*
POD3	2.54 ± 2.69	2.22 ± 1.13	0.554*	2.82 ± 2.82	2.47 ± 1.89	0.474*
POD5	4.82 ± 4.03	3.23 ± 1.86	0.213*	4.90 ± 3.45	4.31 ± 1.94	0.465*
POD7	5.61 ± 1.85	5.17 ± 2.65	0.593^†^	5.08 ± 3.64	5.02 ± 1.37	0.953*
PT ± SD (s)						
Pre-OP	13.34 ± 0.78	13.25 ± 0.67	0.594*	13.24 ± 0.69	13.10 ± 0.81	0.322*
POD1	13.80 ± 1.12	13.98 ± 0.80	0.406*	13.98 ± 1.63	13.89 ± 0.77	0.650*
POD3	13.69 ± 0.88	13.79 ± 0.84	0.631*	13.47 ± 1.91	13.72 ± 1.02	0.301*
POD5	13.56 ± 1.01	13.51 ± 0.83	0.875*	13.22 ± 0.98	13.35 ± 0.86	0.565*
POD7	13.52 ± 0.51	13.55 ± 0.62	0.876*	12.76 ± 1.93	12.89 ± 0.72	0.810*
APTT ± SD (s)						
Pre-OP	35.38 ± 4.29	37.76 ± 9.94	0.228*	35.40 ± 3.78	35.85 ± 3.59	0.496*
POD1	36.11 ± 3.07	36.63 ± 4.05	0.558*	35.49 ± 3.83	35.42 ± 3.22	0.902*
POD3	38.57 ± 4.36	39.76 ± 6.85	0.416*	38.41 ± 4.64	38.52 ± 4.96	0.903*
POD5	37.93 ± 8.42	39.21 ± 4.50	0.494*	38.59 ± 4.81	37.83 ± 5.48	0.570*
POD7	35.14 ± 4.59	37.82 ± 4.14	0.108*	37.80 ± 4.23	36.71 ± 5.57	0.546*

*NFSD* non-fibrinolytic shutdown, *FSD* fibrinolytic shutdown, *FDP* fibrin degradation product, *SD* standard deviation, *Pre-OP* preoperative, *POD* postoperative day, *D-D* D-dimer, *PT* prothrombin time, *APTT* activated partial thromboplastin time

^*^Independent-samples *t* test

^†^Mann–Whitney *U* test

## Discussion

Fibrinolysis is an essential physiological process that maintains hemostatic balance and prevents thrombosis [14]. This process is activated concurrently with coagulation after injury and facilitates the removal of mature fibrin, thereby playing a role in hemostasis to prevent the extension of clots beyond the damaged areas [14]. Pathologically upregulated fibrinolysis, known as hyperfibrinolysis, contributes to insufficient clotting and reduced hemostasis [14]. Hyperfibrinolysis after injury is reportedly associated with increased blood loss, which occurs mainly during the first few hours after injury [15]. During the last decade, several large-scale multicenter RCTs consistently suggested the timely use of antifibrinolytic agents such as TXA soon after the onset of bleeding considering its excellent efficacy in reducing blood loss and decreasing bleeding-related mortality [4, 6]. Currently, TXA is widely recommended as a routine hemostatic agent for all bleeding patients.

Recent evidence from traumatic and bleeding patients, however, has cast doubt on the reasonability of the universal administration of TXA after injury. The fibrinolytic system is not always upregulated after injury but shows a biphasic response (activated or shutdown) [16]. FSD is the most common fibrinolytic phenotype noted after injury, the incidence rate of which has been reported to approximately 59% [17, 18]. Moreover, 70% of patients presenting with FSD could maintain this phenotype for up to 120 h after injury [18]. Owing to the deficiency in fibrinolysis, this phenotype fails to promptly dissolve the excessive clots; it was found to be associated with an increased risk of VTE [11]. Therefore, researchers suggest careful administration of TXA in patients presenting with FSD, for there was nothing to inhibit and may increase VTE risk instead [19].

Existing evidence suggests that the surgical injury associated with TKA significantly activates the fibrinolytic system [20]. Monitoring of the dynamic changes in the D-D level revealed that postoperative hyperfibrinolysis peaks within 24 h after surgery [21]. Thus, sequential administration of TXA within 72 h after TKA may effectively inhibit fibrinolysis activity and reduce HBL [2]. However, recent evidence regarding postinjury fibrinolysis reminds us that the D-D level may fail to act as an accurate parameter for fibrinolytic activity in real time or guide TXA administration.

The fibrinolytic system may be activated in the initial stages after trauma but eventually progress toward a state of shutdown by the time blood is drawn [16, 22]. Given the prolonged residence of D-D in the circulation, assessing the hyperfibrinolysis state on the basis of its level may not be accurate [23]. Notably, TEG, as a comprehensive test comprising both coagulation and fibrinolysis parameters, can evaluate the real-time coagulation and fibrinolytic activities [24]. The seeming paradoxical phenomenon of the elevated D-D levels in injured patients and low fibrinolysis activity measured using TEG is speculated as occult fibrinolysis [22]. Results obtained in the present study demonstrated the existence of occult fibrinolysis in patients who underwent TKA. Furthermore, we found that the fibrinolytic phenotypes after TKA were similar to those after trauma, with FSD accounting for majority of the cases (64.81%). Moreover, although the levels of FDP and D-D were comparable between the two groups, postoperative TXA exerted fairly different effects in patients presenting with different postoperative fibrinolytic phenotypes. Patients presenting with NFSD exhibited significantly lower HBL and ΔHb after postoperative antifibrinolytic therapy, whereas those presenting with FSD did not. This may be explained by the fact that TXA could only improve the fibrin clot strength of patients with NFSD but failed to strengthen that of those with FSD [22]. Our study also showed that the D-D level failed to distinguish patients presenting with FSD or guide targeted therapeutic intervention. Instead, our findings showed that LY30 can be a promising parameter that provides a practicable method for the timely identification of patients who truly require postoperative TXA after TKA. Therefore, given the lack of evidence supporting the administration of antifibrinolytic agents such as TXA in the absence of a target to inhibit, we do not recommend administering TXA after TKA in patients presenting with FSD.

The present study has several limitations. First, this study was retrospective in nature. However, we applied strict inclusion and exclusion criteria to minimize potential bias. Second, we failed to determine dynamic changes in the LY30 level postoperatively because of the retrospective study design. Third, HBL was calculated using widely used formulas based on hematocrit levels, which can be easily influenced by perioperative rehydration strategies. However, the enrolled patients were admitted consecutively and received the same perioperative rehydration protocols. Thus, this should have little effect on the comparisons between groups. Finally, although previous studies have shown increased VTE risk after TXA administration in patients presenting with FSD, we could not obtain sufficient follow-up data to further assess this potential risk.

## Conclusions

The results obtained from this single-center retrospective study report the following findings: (1) patients exhibit various fibrinolytic phenotypes after TKA, (2) postoperative antifibrinolytic therapy after TKA appears to reduce blood loss in patients presenting with NFSD but not in those presenting with FSD, and (3) the LY30 level may be a promising parameter for distinguishing various fibrinolytic phenotypes after TKA and improving the accuracy postoperative TXA administration. However, prospective RCTs are warranted to draw a more robust and clear conclusion.

## Data Availability

The data will be available from the corresponding author upon reasonable request.

## Abbreviations

TKA: Total knee arthroplasty
HBL: Hidden blood loss
TXA: Tranexamic acid
RCT: Randomized controlled trial
FSD: Fibrinolytic shutdown
VTE: Venous thromboembolism
NFSD: Non-fibrinolytic shutdown
LY30: Percentage of clot lysis 30 min after maximum strength
POD: Postoperative day
ABT: Allogeneic blood transfusion
ΔHb: Decline of hemoglobin level
FDP: Fibrin degradation product
D-D: D-dimer
PT: Prothrombin time
APTT: Activated partial thromboplastin time
BMI: Body mass index
TEG: Thromboelastography
